# Community Participation in Health Systems Research: A Systematic Review Assessing the State of Research, the Nature of Interventions Involved and the Features of Engagement with Communities

**DOI:** 10.1371/journal.pone.0141091

**Published:** 2015-10-23

**Authors:** Asha S. George, Vrinda Mehra, Kerry Scott, Veena Sriram

**Affiliations:** Department of International Health, Johns Hopkins School of Public Health, Baltimore, Maryland, United States of America; Harbin Medical University, CHINA

## Abstract

**Background:**

Community participation is a major principle of people centered health systems, with considerable research highlighting its intrinsic value and strategic importance. Existing reviews largely focus on the effectiveness of community participation with less attention to how community participation is supported in health systems intervention research.

**Objective:**

To explore the extent, nature and quality of community participation in health systems intervention research in low- and middle-income countries.

**Methodology:**

We searched for peer-reviewed, English language literature published between January 2000 and May 2012 through four electronic databases. Search terms combined the concepts of community, capability/participation, health systems research and low- and middle-income countries. The initial search yielded 3,092 articles, of which 260 articles with more than nominal community participation were identified and included. We further excluded 104 articles due to lower levels of community participation across the research cycle and poor description of the process of community participation. Out of the remaining 160 articles with rich community participation, we further examined 64 articles focused on service delivery and governance within health systems research.

**Results:**

Most articles were led by authors in high income countries and many did not consistently list critical aspects of study quality. Articles were most likely to describe community participation in health promotion interventions (78%, 202/260), even though they were less participatory than other health systems areas. Community involvement in governance and supply chain management was less common (12%, 30/260 and 9%, 24/260 respectively), but more participatory. Articles cut across all health conditions and varied by scale and duration, with those that were implemented at national scale or over more than five years being mainstreamed by government. Most articles detailed improvements in service availability, accessibility and acceptability, with fewer efforts focused on quality, and few designs able to measure impact on health outcomes. With regards to participation, most articles supported community’s in implementing interventions (95%, n = 247/260), in contrast to involving communities in identifying and defining problems (18%, n = 46/260). Many articles did not discuss who in communities participated, with just over a half of the articles disaggregating any information by sex. Articles were largely under theorized, and only five mentioned power or control. Majority of the articles (57/64) described community participation processes as being collaborative with fewer describing either community mobilization or community empowerment. Intrinsic individual motivations, community-level trust, strong external linkages, and supportive institutional processes facilitated community participation, while lack of training, interest and information, along with weak financial sustainability were challenges. Supportive contextual factors included decentralization reforms and engagement with social movements.

**Conclusion:**

Despite positive examples, community participation in health systems interventions was variable, with few being truly community directed. Future research should more thoroughly engage with community participation theory, recognize the power relations inherent in community participation, and be more realistic as to how much communities can participate and cognizant of who decides that.

## Introduction

### Rationale

The Alma Ata Declaration in 1978 framed community participation as central to primary healthcare [1]. It has also been enshrined as an important principle within rights based approaches to health that has an intrinsic value in and of itself [2]. Since these landmark agreements underpinning community participation, considerable experience has been built regarding it, with ample debate and reflection regarding its definition, rationale and outcomes [3–11]. Community participation can be instrumental as working with communities can help make interventions more relevant to local needs, informed by local knowledge and priorities, and therefore more effective. More fundamentally, depending on the social processes involved, it can also be transformative, helping to empower and emancipate marginalized communities. At the same time, community mobilization without attention to power relations can distort participation from its developmental aims, exacerbate existing patterns of exclusion and further entrench inequities.

Within the last ten years, the role of communities in health systems in low and middle income countries (LMIC) increased in prominence as reviews highlighted the importance of demand side issues [12, 13]. Subsequently, effectiveness trials and systematic reviews demonstrated the health impacts of community health workers [14–16], women’s groups [17, 18], and community initiatives that supported empowerment through micro-finance [19], raising the profile of community-level interventions as an area for further research and investment. Moving beyond improving health practices, service access and intervention implementation, attention to how communities play an essential role in governing health systems through village health committees [20] and other forms of community accountability [21] has also been recently foregrounded in health systems research.

While there is growing consensus on the value of community participation in health systems, there is variation in how communities are defined and understood. While communities are often defined as being geographic, such as in villages or neighborhoods, they are not necessarily territorial, as they can also include social groups united by activities or interests (such as savings or labor groups), and in a range of spaces (whether for example, international or virtual). The Latin word ‘*communitas’* combines the terms ‘with/together’ with ‘gift’, as a broad term for fellowship or organized society. In this sense, communities are constituted by those with a shared social identity; that is of members with the same set of social representations, which are the meanings, symbols and aspirations through which people make sense of their world [22]. These are not purely markers of affinity, but also governed by power relations [23]. In this sense, communities are also heterogeneous and constitute sites of social exclusion [24, 25]. These social conditions are not permanent. Communities are also sites of empowerment, where unequal relations can be challenged [24, 26, 27].

While a defining element in assessing community participation is the level of control or power that communities command in an initiative [28], the terminology that categorizes the processes and conditions by which communities are involved also at times blurred, ranging from mobilization to empowerment. Some view community mobilization as mainly externally driven [29], while others define it as how communities plan, carry out, and evaluate activities on a participatory and sustained basis to improve their health and other needs, either on their own initiative or stimulated by others [30]. Beyond community mobilization lies community empowerment, the expansion of capability to participate in; negotiate with; influence, control, and hold accountable institutions that affect the wellbeing of the community. It is through empowerment that communities gain mastery over their lives and change their social and political environment to improve their health and quality of life [31].

While multiple reviews have argued the value of community participation [3–11], evaluations have largely focused on health outcomes. None assess the extent to which community participation figures in research on health systems interventions. Hence, the purpose of this review is to examine the size and scope of community participation in health systems intervention research in low- and middle-income countries. It is not the intention of the review to provide a comprehensive catalogue of the literature on community participation, as this has already been done by others [3, 4, 9, 29, 32, 33]. Our aim is to review how published health systems research, as one aspect of the health systems policy and research community, is engaging with community participation. The findings are one input towards further understanding and supporting community participation as a part of strengthening health systems research and interventions at community level.

### Objectives

The review sought to understand the extent, nature and quality of community participation in health systems interventions research in LMICs. Participants included community members involved in health systems intervention research in LMICs. As this was largely a qualitative review, specific comparison interventions or populations were not sought and a broad array of study designs were considered eligible, whether experimental, descriptive or exploratory/explanatory. Domains of interest captured by the review include the nature, scale and duration of the interventions that enlisted community participation; its health systems area; type of health conditions and health outcomes derived. With regards to community participation, the review documented extent and depth of community participation; definitions and frameworks used; facilitators and challenges to community participation.

## Methods

A review protocol was developed and shared among team members to guide the review.

### Information sources

We conducted a literature search in June, 2012, of four electronic databases: Pubmed, Embase, Scopus/ Web of Science, Global Health (Ovid). Each database was searched from 2000 onwards for articles containing concepts related to community, capabilities, health systems research and LMICs (Table 1).

**Table 1:** Concepts and associated terms used in literature search.

Concept	Search terms
Community	"Community Networks"[Mesh] OR "Community "[text word] OR “Communities” [text word] OR "Community Health Planning"[Mesh] OR "Community-Institutional Relations"[Mesh]
Capability/ Participation	"Capacity Building"[Mesh] OR “Capability” [text word] OR “Capacity” [text word] OR “Capacities” [text word] OR “Capabilities” [text word] OR “empowerment” [text word] OR “participation” [text word] OR “involvement” [text word]
Health System Research	"Health Services Research" [Mesh] OR "Community-Based Participatory Research" [Mesh] OR "Operations Research" [Mesh] OR OR “Qualitative Research” [Mesh] OR "Evaluation Studies as Topic" [Mesh] OR "Evaluation Studies" [Publication Type] OR "Health Care Evaluation Mechanisms" [Mesh] OR "Program Evaluation" [Mesh] OR "Health Care Quality, Access, and Evaluation" [Mesh] OR "Health Services Research" [Mesh]
LMICs	"Lower-middle-income economies"[tiab] OR “low income economies”[tiab] OR "Developing countries"[mh] OR "developing countries"[tiab] OR "developing country"[tiab] OR "under-developed countries"[tiab] OR "under-developed country"[tiab] OR "third-world countries"[tiab] OR "third-world country"[tiab] OR "developing nations"[tiab] OR "developing nation"[tiab] OR "under-developed nations"[tiab] OR "third-world nations"[tiab] OR "third-world nation"[tiab] OR "less-developed countries"[tiab] OR "less-developed country"[tiab] OR "less-developed nations"[tiab] OR low and middle income countries[tiab] OR lmic[tiab] OR low income country[tiab] OR low income countries[tiab] OR lower income countries[tiab] OR middle income country[tiab] OR middle income countries[tiab] OR lower middle income country[tiab] OR lower middle income countries[tiab] OR “Afghanistan” … Zimbabwe[tiab]

### Article selection

The titles and abstracts of all articles found through the electronic search were combined to form a database and duplicates were removed. In step 1, the titles and abstracts of all unique articles were examined independently by two reviewers, who assessed whether the article should be included or excluded according to our inclusion and exclusion criteria (Table 2).

**Table 2:** Inclusion and exclusion criteria.

Inclusion	Exclusion
Health systems research which examines an interaction of parts (service delivery, information systems, medical products/ technologies, human resources, financing, governance, community/ households) and their interconnections (ideas and interests, relationships and power, values and norms) that come together for a purpose (health)	Basic scientific research, clinical efficacy or effectiveness of treatments/ technologies, measurement and social determinants of population health
Low and middle income country contexts	Editorials
Community level health system interventions are those where communities are substantially involved in their implementation or monitoring and evaluation, ie going beyond initial consultations for design or formative research. Community was defined as people residing together in a geographical area, a village or a township, not inclusive of community based organizations and or local administrators who worked in these geographic areas, but did not reside in them.	Review papers will not be abstracted through the form, but will be reviewed as background material.
English language publication, with American and English spellings	
Peer review journals	
2000 onwards	

Articles on which there was a consensus for exclusion, based on the title and abstract, were excluded automatically. All titles and abstracts assessed as meeting the inclusion criteria by the reviewers and about which the reviewers felt uncertain or disagreed were reviewed by the lead researcher (AG) and discussed with the team to develop consensus on inclusion or exclusion. In step two, full-text versions of all the articles retained in the review were then accessed. These articles were again assessed independently by the review team according to the inclusion and exclusion criteria. All included full-text articles and articles for which there was uncertainty or disagreement were again discussed as a group and assessed by the lead researcher before finalizing the dataset.

### Data collection process and data items

Continuing with step two, an abstraction form was created in Microsoft Excel to facilitate extraction of information from each article on key aspects describing community participation and health systems intervention research as listed under objectives earlier. We assessed study drawing from the Critical Appraisal Skills Program and elements of rigor in health policy and systems research [34, 35]. From these sources, we derived four broad categories in our assessment—sampling, data collection, analysis and trustworthiness. The review team piloted the form independently by abstracting five sample articles. After collective review and discussion, the form was further refined and the researchers reached a consensus on the abstraction process for the remaining articles. The remaining articles were abstracted, with weekly meetings held among the researchers to discuss findings as they emerged, challenges found during the abstraction process and a consensus approach to resolving them. All questions and changes in the abstraction process were documented in a shared document that was reviewed and discussed weekly.

### Analysis

Findings were synthesized using a thematic approach, commonly used to summarize qualitative and quantitative studies in systematic reviews [36, 37]. Articles were revisited multiple times and abstracted findings synthesized into detailed outputs. These were then reviewed and revised by the lead author (AG) in discussion with the team, following a process of constant comparison. After drafting synthesized findings, authors revisited original articles to check their interpretations.

## Results

### Article selection

**Fig 1:**
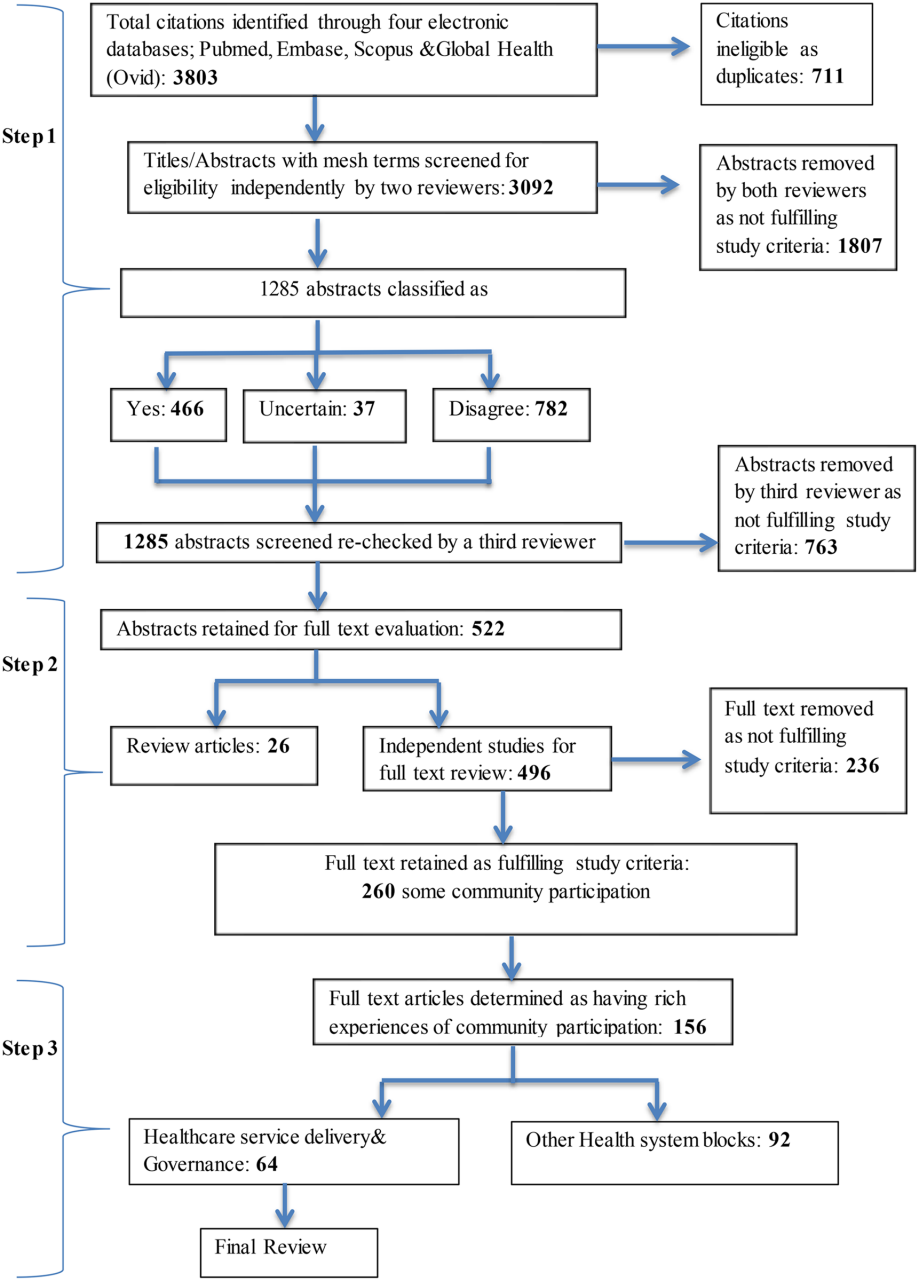
Flow chart detailing article selection

Our search generated 3,803 articles, which after removing 711 duplicates, left a total of 3,092 articles. Next 1807 abstracts where both reviewers agreed on exclusion were excluded. Abstracts where there was disagreement or uncertainty, or which were selected for inclusion (1285) were re-checked and resulted in the removal of an additional 763 abstracts, leaving 522 articles for full-text examination (26 review articles and 496 studies). At the full-text reading stage, an additional 236 studies were excluded after being examined by two reviewers, leaving 260 studies with some level of community participation in health systems research studies. This included articles that aimed to engage communities more fully, but failed to do so (Fig 1 and S1 Table).

### Article characteristics

We assessed geographic location of interventions and first author to assess where and who is publishing research on health systems that involves community participation in LMIC contexts. When authors mentioned two different affiliations, we categorized them by the first affiliation mentioned. Despite the focus on LMIC countries, more than half of first authors were based in high-income countries (58%, 150/260). Among LMIC articles, almost a half focused on sub-Saharan Africa (45%, 117/260), even though just under a fifth were authored by those based in sub-Saharan Africa (19%, 50/260) (Table 3). Very few articles were from LMIC countries in the Middle East and North Africa (n = 4, Yemen and Iran) or in Europe and Central Asia (n = 2, Romania and Kyrgyzstan) and very few spanned multiple regions (n = 4).

**Table 3:** Geographic region of first authors vs. region of intervention.

**Geographic region of first authors**	**Articles with community participation** (n = 260)
Low income	17% (43/260)
Lower middle income	12% (32/260)
Upper middle income	13% (33/260)
High income	58% (150/260)
No information	01% (2/260)
**Geographic region of intervention**	**Articles with community participation** (n = 260)
Sub Saharan Africa	45% (117/260)
South Asia	19% (49/260)
East Asia and Pacific	18% (47/260)
Latin America and Caribbean	14% (37/260)
Middle East and North Africa	02% (4/260)
Europe and Central Asia	01% (2/260)
Multiple regions	02% (4/260)

In terms of study design, just over a half of the articles that included community participation in health systems research interventions were of an explanatory nature (54%, 140/260) and only 7% (19/260) followed a probability design (Table 4). While more articles were either qualitative (37%, 97/260) or combined qualitative and quantitative data (34%, 89/260), a significant number were also purely quantitative (28%, 74/260).

**Table 4:** Study inference.

Study inference	Explanation	Articles with community participation (n = 260)
Probability	Controlled (cluster randomized) trials	7% (19/260)
Plausibility	Concurrent, non-randomized cluster trials	12% (32/260)
Adequacy	Before-after or time-series in program recipients only	16% (41/260)
Explanatory	Can be mixed methods, quantitative or qualitative; focus on how a strategy led to effects on outcome	54% (140/260)
Exploratory	Can be mixed methods, quantitative or qualitative; focus on descriptions and relationships	11% (28/260)

### Synthesis of results for all health systems research articles with community participation

In this section, we review how communities participated in interventions, who in communities participated and the distribution of articles across health systems domains and health conditions.

#### Extent of community participation: How communities participate?

The extent of community participation in health systems research interventions was assessed across five different elements, depending on whether communities were involved in: (1) identifying and defining the problems addressed; (2) identifying and defining the interventions developed to address those problems; (3) implementing interventions; (4) managing resources for the interventions; and/or (5) monitoring and evaluating interventions. To be included in this review, articles needed to have community participation in at least one of the above five elements (Table 5).

**Table 5:** Nature of community participation.

Nature of community participation (CP)	Articles with CP (n = 260)
Identifying and defining problems	18% (46/260)
Identifying and defining interventions	50% (131/260)
Implementing interventions	95% (247/260)
Managing resources for intervention	31% (80/260)
Monitoring, evaluating interventions	24% (63/260)

Of those articles that had some degree of community participation in the health system intervention under investigation, almost all detailed community participation in implementing interventions (95%, 247/260). Very few were involved in the strategic decisions that framed the research by identifying and defining the problems that needed to be addressed (18%, 46/260), although just over half were involved in identifying and defining interventions (50%, 131/260). Fewer articles detailed community participation in terms of managing resources (31%, 80/260) or monitoring and evaluating (24%, 63/260). Only a minority involved communities in 3 steps (55/260, 21%) or in 4 steps (12%, 31/260), with only 4 involving communities in all 5 steps.

We combined our assessment of the number of elements with the level of detail available on community participation in the article to categorize articles as having “rich” community participation. Those categorized as rich participation largely correlate with the increasing number of elements, but not exactly (Table 6). For example, articles that may have only supported community participation in one or two elements of the intervention but provided a rich description of this participation whether positive or negative where included, rather than those that had more than one element but with little description detailing what this meant for the communities involved.

**Table 6:** Number of elements of community participation.

Number of community participation (CP) elements	Articles with CP (n = 260)	Articles with rich CP (n = 156)
	*Column subtotals*	*Row subtotals*
CP in 1 of the 5 elements	33% (86/260)	22% (19/86)
CP in 2 of the 5 elements	32% (84/260)	57% (48/84)
CP in 3 of the 5 elements	21% (55/260)	98% (54/55)
CP in 4 of the 5 elements	12% (31/260)	100% (31/31)
CP in all 5 elements	2% (4/260)	100% (4/4)
Total	260	60% (156/260)

#### Extent of gender analysis: Who in communities participates?

Among those articles with rich community participation, just over half, 54% (84/156) disaggregated by sex. However, those that did present information disaggregated by sex, did so regarding mainly related to background data sources or population health outcomes. Extremely few articles detailed intervention participation by sex [38–40].

Almost a third, 32% (50/156), of articles targeted women or men or focused on sex specific health conditions. A large number of these articles focused on sexual, reproductive, maternal and child health issues, primarily focusing on women as beneficiaries. Only one article targeted men in participatory way by supporting father’s clubs to promote child health [41].

Of the 106 articles with rich community participation that did not target women or men or their sex specific health issues, 28% (30/106) did not discuss gender in any way, 35% (37/106) mentioned gender in passing (one or two sentences), and only 28% (30/106) discussed gender issues substantively.

#### Health systems domains

We assessed which health systems domains had interventions that involved community participation, and identified eight different fields: (1) health promotion, (2) inter-sectoral, (3) service delivery, (4) governance, (5) supply chain management, (6) financing, (7) human resource management and (8) information systems (Table 7).

**Table 7:** Extent of community participation (CP) across health systems domains.

Health systems domains	Articles with CP (n = 260)	Articles with rich CP (n = 156)
	*Column subtotals*	*Row subtotals*
Health promotion	78% (202/260)	63% (128/202)
Inter-sectoral	35% (90/260)	71% (64/90)
Service delivery	30% (77/260)	69% (53/77)
Governance	12% (30/260)	80% (24/30)
Supply chain management	9% (24/260)	83% (20/24)
Financing	7% (19/260)	68% (13/19)
Human resource management	7% (18/260)	50% (9/18)
Information systems	3% (7/260)	29% (2/7)

Involving communities in health promotion was most common, with 202 of the 260 articles (78%) included in the review having communities participating in this domain. However, only 63% (128/202) of the articles that involved communities in health promotion included rich experiences of community participation. Most of the health promotion articles in our review included community participation in implementing the intervention, but were less likely to have communities defining the problem that needed to be addressed, defining the intervention in question, managing resources for it or monitoring/evaluating its results.

Involving communities in governance and supply chain management was not very common (only 12% (30/260) and 9% (24/260) respectively), but when it occurred it was highly participatory with 80% (24/30) and 83% (20/24) of the articles in these respective domains being classified as having high participation. Within the domain of governance, many of the articles described how communities were engaged in decision making regarding the intervention, their involvement in health planning processes or supervision of services. With regards to supply chain management interventions, half of the interventions included related to community-directed treatment for various communicable diseases, which involved communities in implementing the intervention and often in developing the intervention, managing resources for it and at times monitoring it.

Information systems was the health system domain with the fewest articles 3% (7/260) and also the least participatory 29% (2/7). These interventions included studies on community-based surveillance systems for specific health conditions or treatment programs [42–44], community engagement in social audits or quality improvement [45, 46] or the challenges of engaging community participation in information systems in post-disaster situations [47]. In most of these articles, coordination, management and action on data was undertaken by local government bodies or NGOs and not directly by community members themselves, with the exception of du Mortier et al. (2005) and Heinonen et al. (2000) [46, 48].

#### Types of health conditions

We assessed which types of health conditions were addressed through interventions that involved some community participation. Community participation was most frequently observed in interventions targeting HIV, followed by articles pertaining to other infectious diseases and the environment. For example, a large number of articles documented community directed treatment with ivermectin (CDTI) for controlling onchocerciasis and lymphatic filariasis, and other community-directed interventions (CDI) for insecticide-treated bed nets, etc. However, after aggregating across health conditions, relatively similar proportions of articles focused on reproductive and child health (38%, 99/260) and HIV, Malaria and TB (32%, 84/260) when compared to those focusing on other health conditions (29%, 76/260) and broader determinants of health (27%, 69/260) (Table 8).

**Table 8:** Extent of community participation across health conditions.

Type of health condition	Articles with CP (n = 260)	Articles with rich CP (n = 156)
	*Column subtotals*	*Row subtotals*
**Reproductive and child health**	**38% (99/260)**	**47% (47/99)**
Maternal	13% (34/260)	50% (17/34)
Under five/Newborn	13% (35/260)	51% (18/35)
Family planning	6% (15/260)	27% (4/15)
Other sexual and reproductive health	6% (15/260)	53% (8/15)
**HIV, Malaria and TB**	**32% (84/260)**	**52% (44/84)**
HIV	25% (64/260)	55% (35/64)
Malaria	4% (11/260)	55% (6/11)
Tuberculosis	3% (9/260)	33% (3/9)
**Other conditions**	**29% (76/260)**	**62% (47/76)**
Other infectious diseases	18% (46/260)	59% (27/46)
Non communicable disease	12% (30/260)	67% (20/30)
**Other broader health determinants**	**27% (69/260)**	**67% (46/69)**
Environment	15% (40/260)	63% (25/40)
Broader health issues, primary care	11% (29/260)	72% (21/29)

### Synthesis of results for service delivery and governance articles with rich community participation

Of those with rich community participation we did further analysis on the 64 that involved service delivery and governance in health systems research due to our interest in service delivery and because governance is the health systems domain most likely to have insights on power, which lies at the heart of community participation. Of these, 53 had service delivery elements and 24 had governance elements, with 13 articles with both service delivery and governance elements. In this section, we first review the quality of the studies, the nature and scale of the interventions and their implications for health. We then focus on how the articles described community participation in terms of the definitions and theoretical frameworks used, the balance of power described, and the factors that facilitated or challenged community participation.

#### Quality of study design and analysis

Despite being lenient in interpreting responses regarding quality of study design and analysis, several aspects of study quality were glaringly deficient. No one article fulfilled all the elements expected of research articles independent of study design. Basic elements related to describing study area and selection, data sources and collection, and triangulation across data sources were listed by at least 70% or more of articles. Nonetheless, ethics statements were only found for 25% (16/64) of articles and limitations acknowledged in only 36% (23/64) of articles. Considering that these articles included rich examples of community participation with implications for how power relations were addressed, it is striking that respondent validation was only found in 17% (11/64) of articles and reflexivity in 8% (5/64) (Table 9).

**Table 9:** Study quality.

Elements of study quality	Articles with rich community participation in service delivery and governance (n = 64)		
	Yes	Partial	No
	**Sampling**		
Study area described	97% (62/64)	0% (0/64)	3% (2/64)
Study area selection explained	72% (46/64)	2% (1/64)	27% (17/64)
Sampling criteria mentioned	59% (38/64)	0% (0/64)	41% (26/64)
Non-participation rates	16% (10/64)	0% (0/64)	84% (54/64)
	**Data methods**		
Data sources listed	83% (53/64)	2% (1/64)	16% (10/64)
Data collector training/ piloting mentioned	25% (16/64)	2% (1/64)	73% (47/64)
Data collection described	73% (47/64)	2% (1/64)	25% (16/64)
Supervision mentioned	5% (3/64)	0% (0/64)	95% (61/64)
Ethics statement mentioned	25% (16/64)	0% (0/64)	75% (48/64)
	**Analysis**		
Methods stated	67% (43/64)	8% (5/64)	25% (16/64)
Limitations stated	36% (23/64)	0% (0/64)	64% (41/64)
	**Trustworthiness**		
Triangulation by data source	73% (47/64)	0% (0/64)	27% (17/64)
Triangulation by respondent	61% (39/64)	0% (0/64)	41% (25/64)
Respondent validation stated	17% (11/64)	0% (0/64)	83% (53/64)
Reflexivity stated	8% (5/64)	0% (0/64)	92% (59/64)

#### Nature of the interventions

The service delivery interventions focused mainly on supporting capacity building and partnerships across various stakeholders for community level programs. These included supporting CDTI, but also community-directed programs related to managing other commodities to address lymphatic filiariasis [49], malnutrition [50], anaemia [51], misoprostol [52], and malaria, vitamin deficiency and tuberculosis [53]. Other programs included community-based initiatives supporting safe motherhood and women [54–56], multi-sectoral basic needs [48], family health at the provincial level [57], or in urban contexts [58], urban provision of emergency care [59], or HIV/STI prevention [60, 61]. Other types of participatory service delivery interventions involved community-based care models in conjunction with: training professional health providers [39, 62, 63]; women’s groups [64–67]; drug revolving funds/ risk pooling mechanisms [68–70]; and community transport initiatives [54, 55, 71]. A few articles detailed community research processes that facilitated better understanding of marginalized groups such as injecting drug users [72] or parents’ perceptions of children’s fever for malaria programs [73]. Several programs worked with community members to build their capacity to become providers themselves and better support community needs [74–77].

Governance interventions ranged from quality improvement and supervision initiatives [46, 78, 79], to community monitoring efforts [80, 81], and user associations, health committees and other mechanisms to facilitate public participation in planning and health service oversight [82–90].

#### Scale and duration of interventions

Of the 64 articles with rich experiences of community participation service delivery or governance in interventions with service delivery and governance elements, all except three included information on the intervention’s location and scale. Interventions that supported rich community participation ranged from very localized efforts in one village, city or township, to those in various localities, whether in one district or more, with no discernable pattern. Five out of the seven articles detailing national level experiences were those where the MOH either adopted decentralization or enhanced public participation through committees [81, 84–86, 91]. Four out of the five multi-country interventions pertained to community-directed treatment for onchoceriasis.

A few articles documented experiences of community participation that was scaled up or implemented in phases, reflecting flexibility in adapting processes to fit changing needs and local contexts [66, 92, 93]. For example, in Bangladesh, women’s groups were scaled up from 162 to 810 groups, with adaptations related to the schedule and content of group meetings to emphasize participation of women in reproductive age and especially pregnant women [66].

Although fourteen articles failed to report the duration of the projects involved, a sizeable proportion of articles detailed interventions that lasted between one to five years (n = 29/65, 45%). Projects that were more than five years but under 10 years were often part of government decentralization initiatives. Similarly, of the six that continued beyond ten years, half were national programs or reforms [83, 86, 94] undertaken by the Ministry or Department of Health and thus institutionalized into existing health systems.

In articles that described institutionalization, integration was commonly ensured by either incorporating intervention specific cadres and components within existing health systems [55, 56, 81, 95], other government and religious organizations [59, 68] or by building onto pre-existing structures [50, 64, 77, 80, 91, 92, 96]. In other instances, interventions were either embedded within national/, local or village level governance structures [51, 55, 82, 87, 92, 94] or managerial and decision making processes through formation of associations or executive committees [53–55, 77, 90].

Extremely few articles were able to assess sustainability of interventions after the project ended. The strongest example is a maternal health project in Tanzania that supported village health workers and community financing for emergency transport. It measured sustained utilization of maternal health services and knowledge about danger signs of pregnancy six years after its completion [55].

#### Health and health care effects

With regards to changes in health and health care, among the service delivery and governance articles with rich community participation, expanding service availability was the most documented health outcome, with nearly 63% (40/64) of the articles indicating increasing service availability through community-directed treatment intervention [97], construction of new facilities [87], or an increase in services provided by particular cadres [98]. Similarly, just over half of the articles, 52% (33/64), noted improvements in accessibility, in terms of both geographic accessibility by increasing the availability of services, enabling care closer to households or improving access to transportation, as well as financial accessibility through free-of-cost services [59], elimination of user fees [93] or the development of mother and child funds [64]. Nonetheless, these increases were not always well measured [48, 90] or at times were not enough to overcome systematic or structural challenges, such as those related to supply chain or geographical remoteness [58, 75].

Over half of the articles, 57% (37/64), detailed improvements with regards to acceptability, ranging from familiarity through peer involvement to service satisfaction and facility cleanliness. For example, the recruitment of community health workers allowed for interventions to become more culturally acceptable to community members [40, 96, 99]. Challenges included mistrust or abuse by providers [58, 61, 64, 76] and culturally inappropriate gender of providers [59].

In contrast, fewer articles, 23% (15/64), focused on improving quality of services and fewer still reported success in this aspect. Positive experiences included the role of communities in developing action plans with providers [78] and monitoring commodities, including drugs [80]. Health committees were involved in improving pharmaceutical management in India, [51] and the Democratic Republic of Congo [46]. However, challenges such as shortages in supplies and drugs, poor inter-personal skills of providers with patients, lack of training of providers and perceived lack of skill and lack of trust of providers were noted [58, 65, 76].

Changes in health behavior were noted in 33% (31/64) and in morbidity or mortality in 23% (15/64) of the studies. Community members played a role in improving lifestyles and supporting health care seeking. Some articles documented activities that supported behavior change, but did not measure whether this happened [90, 98, 100]. Challenges to achieving health outcomes included ‘fears’ or beliefs that services might negatively impact individuals, such as side effects of vaccinations or drugs [88, 101], as well as conservative gender norms [38]. Methodologies of those studies that did report a decrease in morbidity or mortality range from analyses of project evaluation data and case studies to randomized control trials. Only the women’s group interventions from Nepal and India had study designs where declines in mortality could be statistically inferred [64, 67, 102].

#### Use of definitions or theoretical frameworks

Authors used a diverse set of definitions and frameworks in explaining the concept of community participation. Of the 64 articles related to governance and service delivery, only 16 provided a definition or framework of community participation. Five articles used Rifkin’s concept of the five stages of community participation in health [29, 52, 68, 75, 84]. Other definitions and frameworks referenced by authors include Zakus and Lysac [63, 94, 103], Arnstein’s ladder of community participation [38] and Ugalde’s definition of symbolic participation [82, 104]. The remaining five articles defined community participation in their own words, or in a manner that illustrated how participants constructed the concept for themselves [58, 59, 63, 99, 100].

Articles also discussed community participation as a part of related concepts such as social capital [96]; community development movement [98]; and primary health care [99]. In addition to or separately from references to community participation, articles also specifically referenced concepts such as empowerment [63, 74, 84, 96, 98], sustainability [57, 58, 105], and collaborative or community-based participatory research [72, 80, 106]. Four articles, including two focused on women’s group interventions, explicitly mentioned Freire’s concepts of critical consciousness and/or empowerment education [65, 67, 74, 96].

#### Balance of power

Although power is a central part of understanding community participation, only five studies mentioned power or control [40, 52, 60, 91, 93]. We drew on Rifkin’s work to categorize the depth of community participation as either community mobilization, collaboration or community empowerment [107] depending on the level of community participation, its scope of influence, view of health and the balance it drew between communities and professionals (Table 10). Most articles described community participation processes that resembled collaboration 89% (57/64), with fewer describing either community mobilization or community empowerment, each 53% (34/64).

**Table 10:** Balance of power and the continuum of community participation.

Level	Scope of influence	View of health	Balance between communities & professionals
Community mobilization	Medical	Absence of disease	People do what the professional advises
Collaboration	Health services	Physical, mental and social well being	Communities contribute time, materials and/or money, but with the professionals defining needs
Community empowerment	Community development	A human condition	Planning and managing health activities by the community using professionals as resources and facilitators.

About half of the articles that described community mobilization in service delivery and governance interventions focused on raising community awareness around a specific health issue through drama presentations, video clips, education sessions, picture cards etc. In addition, a few interventions trained community volunteers to raise awareness and conduct health promotion through household visits or counseling [50, 54, 55, 60, 87]. Project stakeholders also conducted community level meetings to sensitize communities and enlist their support for the intervention. Yet, only a couple of articles described feedback sessions with communities; mostly about the results of baseline surveys stressing the importance of an impending health problem [51, 73]. Overall, many articles did not detail who was in charge of the participatory interventions or who set the agenda defining interventions.

About a third of the service delivery and governance articles that described collaborative forms of community participation involved communities in planning, evaluation and supervision of the intervention. Many articles described professionals working with communities in recruiting and training community volunteers to implement service delivery or governance interventions. In other cases, communities mobilized resources such as funds [55, 59, 72, 87, 98, 101], or materials and supplies [53, 59, 77, 78, 108]. In one instance, the development of a protocol to manage mental illnesses [92] illustrated the meaningful and substantial contribution by communities to addressing a critical community problem.

In almost half of the articles that we classified as describing community empowerment, communities became skilled in identifying and prioritizing problems; devising action plans; and implementing, monitoring and evaluating the plans. This was a skill common to women’s group interventions where women learnt problem solving techniques through participatory learning and action cycles. In Nepal, groups went through a cycle of problem identification, planning, implementation and evaluation to initiate and implement strategies as stretcher schemes, revolving funds for obstetric or newborn emergencies, and making and distributing clean home delivery kits to counter maternal and newborn health issues [65]. In almost a third of the articles, communities were also found to actively engaged in either managing budgets [59] or raising funds for the continuation of the intervention [48, 51, 64, 68, 96].

Apart from women’s groups, CDTI interventions also successfully empowered communities to make local management decisions [40, 53, 99, 101, 109, 110]. CDTI interventions required that communities assume full ownership for control of onchoceriasis and it was found that communities managed to do so by selecting and supporting ivermectin drug distributors, collecting supplies of drugs, and determining the period, place and method of drug distribution.

As important as it is to try and discern the balance of power involved in community participation efforts, these categorizations are not mutually exclusive, as a few articles (n = 11) had all three levels of community participation. It was commonly observed that even when a community was empowered to implement and manage a program; it still collaborated and followed the lead of an agency or health service personnel for some activities. With CDTI in Uganda, communities assumed responsibility of selecting community drug distributors, venue, method of drug distribution, procurement and storage of drugs and were able to execute managerial decisions with minimal external interference. However, communities collaborated with personnel from district health facilities in supporting health education sessions, as well as training and supervising community drug distributors [109].

Some articles detailed partially successful initiatives to empower communities [38, 50, 54, 75, 89]. For example, in Tanzania, a service delivery intervention sought to empower communities to manage maternal emergencies through participatory development of community-based plans for emergency transport [54]. The author found that though the project built community capacity to develop transportation plans and manage resources, subsequent evaluation revealed that none of the target villages were at the level where they could develop and execute their own plans. More than half of the communities were at the level where they endorsed and cooperated in promoting the community based reproductive health program and the rest where at the level where the communities’ role continued to be of advice and consent. In other projects, attempts to foster deeper community participation failed as the intervention came under the control of few influential people such as local leaders and officials, rather than the broader community.

#### Facilitators and challenges to supporting community participation

Almost all 64 service delivery and governance articles with rich community participation documented both facilitating and challenging factors.

At the individual level, many intrinsic elements of motivation supported the willingness of community members to engage with participatory processes: professional and personal growth, respect and recognition [39, 55, 58, 101]; a sense of confidence and ownership [73, 75, 79, 84] and the development of leadership skills and knowledge [55, 79]. In contrast, lack of appropriate levels of training, skills, education and interest [64, 72, 75, 86]; as well as insufficient information regarding roles and responsibilities were commonly stated barriers to effective participation [49, 58, 74, 81, 82, 89, 91]. In four articles, it was observed that financial compensation was not a necessary entity for initiating participation, but was needed for continued performance of volunteers [68, 70, 99, 105].

Many articles noted that even adequately trained and motivated individuals were unable to effect action if they lacked community support or if community members did not trust them or appreciate the activities they were carrying out. Trust building mechanisms, such as the democratic selection of community-based volunteers by community members, were important [40, 50, 53, 76, 101, 108, 109, 111]. When selection was managed by traditional kinship structures [109, 111, 112], this aided community participation and intervention effectiveness within the kinship group, but led to continued exclusion of others not part of that group.

Another contextual factor supporting community participation was concurrence with prevalent cultural norms [56, 65, 73, 101, 111] or fit with local environment and needs [50, 51, 67, 71, 80, 92]. A community-based safe motherhood intervention strategically trained and included men as outreach workers raising awareness about obstetric complications [56]. This strategy facilitated community participation because in it accounted for the influential role of men in Tanzania. Other interventions involving pagoda members [68, 88], pre-existing church groups [77] and Sikh community structures [87] engaged with pre-existing religious institutions to motivate communities to participate.

Besides contextual factors, communities were more likely to participate across the breadth of interventions if they either perceived or experienced an intervention to be beneficial [49, 51, 53, 101]. Other factors that led to communities organizing around a health issue were a history of shared struggle such as displacement or conflict [108], communities posed with geographical challenges [55, 70] or situations leading to self-help and voluntarism due to the adversities faced [54, 93, 108, 113].

Community participation was also shaped by characteristics of people outside the community. A positive perception and enthusiasm for a community’s contribution among health workers and project staff virtuously fed back to enhance community participation [48, 61, 69, 89, 93, 98]. Conversely, lack of trust and support from external stakeholders, devaluation of community input and lack of confidence in their abilities inhibited community participation [53, 58, 62, 70, 76, 100, 101]. At times, the negative perceptions of communities held by external actors were exacerbated by contextual features such as vertically oriented health systems with top down approach to decision making and dominance by medical professionals [62, 74, 75, 98].

Several processes were found to support community input at various stages of program development, implementation and evaluation. For example, participatory research and social mapping were identified as key processes to developing consensus between different stakeholders and promoting program acceptability [92, 106]. Representation through community-based organizations, health committees and discussion forums also lent voice to communities, enhanced ownership and successfully supported community participation. At the same time, in certain contexts, health boards, health committees and district health management teams meant to promote wider community participation either made no concerted efforts to collaborate with communities or were more symbolic than practical [100]. Six articles noted the importance of transparency, open communication and increased accountability to communities as critical to supporting community participation [50, 55, 69, 72, 77, 98].

One of the most significant contextual factors resulting in increased community participation included changes in political inclination to devolve responsibilities to local people (11 out of 29 articles that discussed contextual factors), through for instance government reforms and policies mandating the inclusion of communities in program planning and implementation. For example, the Social Reform Agenda and Local Government Code in the Philippines enabled increased community participation in local governance through devolution of powers from Departments to local governments [48]. While several articles did document broad contextual factors whether related to authoritarian politics in Cambodia [88] or Apartheid in South Africa [39, 106], better documentation and research is required to understand when and how such contexts hinder or support health systems interventions that hinge on community participation.

## Discussion

### Summary of evidence

In terms of article characteristics, the majority of articles documenting community participation in health systems intervention research focused on sub-Saharan Africa, in stark contrast to lead authorship of those articles. This may reflect a type of publication bias, wherein those at national and community level may have less interest in publishing in international journals, due to their political commitments and career incentives. Regardless of the reasons, the contrast reflects the skewed nature of global health research [114, 115]. UNESCO’s 2010 Science Report indicates that 62% of researchers and 75% of scientific research publications were from high income country institutions [116]. While the importance of building research capacity in LMICs was emphasized in the 1974 World Health Assembly and re-affirmed since [117–119], to our knowledge, systematic reviews in general, let alone those on community participation [3, 5–7, 11, 120], examine this inequality.

While there is a mix of purely qualitative, quantitative or mixed methods studies in our review, most were explanatory in nature, with very few using probability or experimental designs to assess health effects. The quality of studies, irrespective of their study designs, was variable. While it may not be possible for any one study to list all the elements of good quality design that we detailed, the paucity of studies that listed their sampling criteria, study participation rates, ethics approval, study design limitations, respondent validation or reflexivity is of serious concern.

Other reviews on community participation have also noted the paucity of experimental designs testing the effectiveness of community participation [11, 120], others have called to attention the lack of process evaluations [6] and qualitative research [5] to more clearly examine how community participation contributed to the health outcomes attributed to it. Rifkin argues that the difficulty in finding such an effect is due to mistaking community participation as an intervention, rather than a social process requiring alternative evaluation designs [3]. Considering the variable study quality of many of the articles in our review, and in those of others [120], and the challenge of assessing community participation, due to its complex, context-specific and contested nature, better quality research to further understand the nature of community participation is required and efforts claiming to assess its effectiveness without such understanding treated with caution.

Looking across health systems domains, the largest number of articles supporting community participation were those detailing health promotion interventions. Yet they were not the most participatory, as many of the interventions were didactic in nature, with the problems targeted and the design of the health promotion interventions determined by those outside the community. Articles that focused on governance and supply chain were less common, but more participatory, as governance interventions tended to focus on social accountability, and many of the supply chain articles were about community drug distribution systems. Articles that supported community participation in information systems were the least common and least participatory, reflecting how much control over information is retained by project personnel outside of communities. To our knowledge, no reviews have assessed the ability to support community participation in one health systems domain vs. another. At least one article that contrasted a participatory youth peer education effort vs. a participatory youth health care service, found that the latter by providing tangible benefits and by providing an accepted bio-medical context facilitated empowering outcomes more easily, yet was more modest in the empowering aims it had [96]. Further understanding of how the specific characteristics of each health system domain may influence community participation is required.

Although there were a large number of articles related to HIV interventions that supported community participation, when aggregated into broader categories, there was an even spread between reproductive, maternal and child health areas; TB, malaria and HIV; other health conditions; and broader determinants of health. This partially reflects the large contribution HIV has played working with communities on health promotion or preventive initiatives, but also reflects how community participation as a principle is relevant and actively used by initiatives across all kinds of health conditions.

Closer examination of the subset of articles reporting rich community participation in service delivery and governance, revealed that while there was a diversity of scale and duration of interventions, those that were at national scale and lasted more than five to ten years were often part of government programs, including decentralizing initiatives. With regards to health and health care, most of these articles with rich community participation in service delivery and governance documented improvements in service availability, acceptability and accessibility. Very few attempted to improve quality of care and with any success. Although several articles detailed effects on health behaviors, morbidity or mortality, most, barring a few exceptions, were not designed methodologically to credibly assess changes in health outcomes. This partially reflects the health systems focus of the subset of articles we focused on (service delivery and governance), but also the weakness in study quality that we noted earlier.

With regards to the extent of community participation detailed, even with only including articles that had more than just nominal community involvement, most of the articles mainly detailed community participation in *implementing* interventions. Very few engaged communities in identifying or framing the problems to be addressed and relatively few engaged communities in managing resources or monitoring and evaluating interventions, although just over half did involve communities in the design of interventions. Few articles involved communities in 3 or more steps of the health systems intervention, with only 5 involved in all, suggesting significant shortfalls in the participatory intent of most articles. Others have noted that interventions supporting community participation often achieve less community participation than originally planned [120]. In another review, among the 9 studies detailing high levels of community participation in high income countries, despite democratic and shared decision-making and community initiation or majority seats in some instances, financial control and financial decision-making was retained by outsiders [7].

Also notable was the lack of information on the social characteristics of the community members involved, as just over half of the articles disaggregated details about their work by sex. Of those articles that did include men and women among their participants, more than a quarter failed to discuss gender in anyway, in contrast to the more than a quarter that did. Other have also found inadequate information about who is included in participatory initiatives at community level [7]. This is striking considering the known social hierarchies within communities.

Very few articles cited a definition or framework for community participation and only five studies mentioned power, a central element of participatory processes. Similarly only four mentioned Freire, despite his seminal role in advocating for community empowerment and critical consciousness. At the same time, the great majority of articles in our review described community participation processes that resembled collaboration, with just over half describing either community mobilization or community empowerment. This partially reflects our selection of articles that had more than nominal community participation, but also a potential publication bias towards studies where external agents had resources for evaluation, rather than instances that were more community led and financed [6]. It also reflects how fluid some of these nuances are, as the balance of power within projects can change over time, as does the character of the internal and external stakeholders, along with their motivations for supporting community participation.

Facilitating and challenging aspects of community participation were documented by all the articles to some degree. At the individual level, community members are motivated to engage for a variety of intrinsic reasons, although financial support cannot be ignored for long term continuity. Community level support and trust was indispensable and facilitated when communities’ perceived interventions to fit with their needs and adapted to their context. External linkages particularly to the health care system was seen as important, and at times inhibited by broader factors. Institutional processes that facilitated trust, transparency and communication were central. Contextual factors supportive of community participation included legal reforms or policies leading to decentralization inclusive of communities, as well as specific political histories and engagement with social movements. Others have also emphasized multiple layers of factors that influence community participation [11], most particularly the importance of trust, acceptance and long term partnerships [7].

### Limitations

The literature documenting health systems interventions at the community level is large, but with no standard definition or reporting guidelines on how to describe community participation, it took time to identify those articles that documented some kind of community participation versus those that implemented at the community level with nominal community involvement. Others have also cited this challenge [6]. Furthermore the paucity of description in many articles made understanding central aspects of community participation, in terms of who from communities were involved; the balance of power; when, how were communities involved and with what consequences, challenging. Studies may have considered and measured many of the elements central to this review, but not published them in the articles that were included in our review. Articles with richer descriptions may have been over-represented in the qualitative analysis only because they provided information. While we did present quantifications to characterize the literature, a significant portion of the decision-making, abstraction and interpretation is subjective. Throughout the review, we therefore not only convened regular group discussions to evaluate our understanding of the subject, but also documented our deliberations.

## Conclusions

While several positive examples of community participation exist in the review, there are important elements of caution also highlighted. Despite the history and value of community participation, there remains a lack of common understanding of concepts, motivations and social processes underpinning community participation. Many articles are largely under-theorized and not self-critical, with few making reference to definitions or frameworks. While this may not seem relevant to either the social transformation or utilitarian goals motivating community participation initiatives, it can help to explain the assumptions underpinning the type of community participation project supported and clarify expectations about the extent of change envisaged and the inputs required to realize it at multiple levels. Apart from tangible inputs and skills, relational issues related to trust and transparency are essential. In addition to the role of strong champions, broader structural policies that create supportive spaces for community participation are also important.

Projects supporting community participation were found across all health system domains and health conditions, across varying contexts and scale, but this does not mean that maximum community participation across all elements of project management is the ideal. Many articles reported involving communities and supporting community participation, most were collaborative in nature with a balance between community and outsiders directing the intervention, with very few being truly community directed. Whether this is appropriate or not depends on the context, and most importantly what communities themselves want with regards to their role in health systems interventions. Maximum participation without delegation of resources or democratization of power, may marginalize those communities and members that can least afford to participate. Yet few articles discussed power or control in developing and implementing their participatory interventions with communities. Understanding, negotiating and contesting power remains a foundation to be laid with health systems researchers, health systems interventions and societies.

## Supporting Information

S1 File PRISMA checklist. (DOC)
